# Supplementary material for: Cross regulation in a three-component cell envelope stress signaling system of Brucella
Source: bioRxiv. 2023 Oct 11:2023.04.15.536747. Preprint. [Version 2] doi: 10.1101/2023.04.15.536747 (PMC10592609; doi:10.1101/2023.04.15.536747)
Supplement: 1 [file NIHPP2023.04.15.536747V2-supplement-1.pdf]

bioRxiv preprint doi: https://doi.org/10.1101/2023.04.15.536747; this version posted October 11, 2023. The copyright holder for this preprint (which was not certified by peer review) is the author/funder, who has granted bioRxiv a license to display the preprint in perpetuity. It is made available under aCC-BY 4.0 International license.

Supplemental Figures

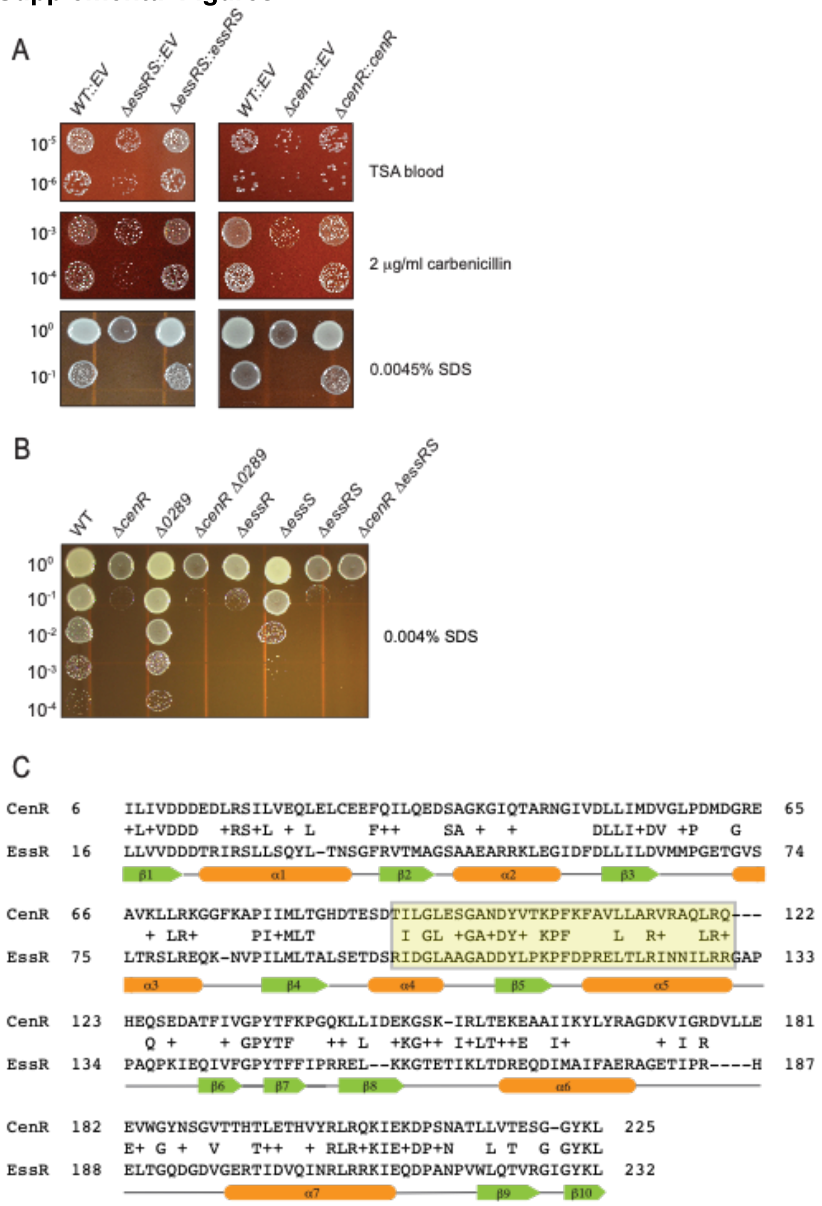

Figure S1. Genetic complementation of DessRS and DcenR mutants; SDS sensitivity assay at a reduced SDS concentration, and CenR-EssR sequence alignment. (A) Genetic complementation of Δ*essRS* and Δ*cenR* carbenicillin and SDS phenotypes. Complementing copies of the deleted genes were inserted into the ectopic *glmS* locus using Tn7. Dilution plating experiments were repeated at least three times for all strains. One representative experiment is shown. (B) SDS resistance phenotypes of strains harboring in-frame unmarked deletions (D) of *B. ovis* TCS gene loci *BOV_1929* (*cenR*), *BOV_0289*, *BOV_1472* (*essR*), *BOV_1473* (*essS*), alone and in combination show a larger dynamic range at an SDS concentration of 0.004% compared to 0.0045% (as presented in Figure 1). Dilution plating experiments were repeated at least three times for all strains, and one representative experiment is shown. (C) Amino acid sequence alignment of CenR and EssR. Amino acids highlighted in yellow show the primary structure of the α4-β5-α5 protein-protein interaction interface predicted by AFComplex2 (see Figure 5C). Protein secondary structure is presented above the alignment (helix in orange; strand in green).

bioRxiv preprint doi: https://doi.org/10.1101/2023.04.15.536747; this version posted October 11, 2023. The copyright holder for this preprint (which was not certified by peer review) is the author/funder, who has granted bioRxiv a license to display the preprint in perpetuity. It is made available under aCC-BY 4.0 International license.

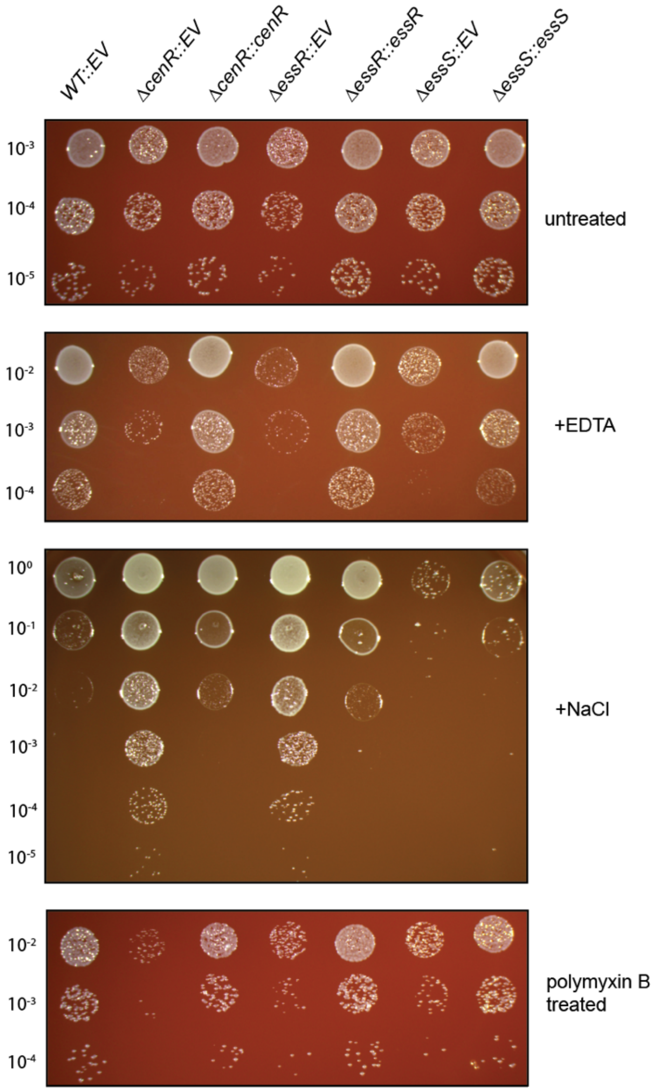

Figure S2: *cenR, essR,* and *essS* contribute to cell survival in the presence of diverse envelope stressors. Strains harboring in-frame unmarked deletions (D) of *cenR, essR, essS*, carrying integrated empty vectors (EV) or genetic complementation vectors (::*gene locus number*) were plated in log_{10} dilution series on plain TSA blood agar (untreated), TSA-B containing 2.75mM EDTA (+EDTA), TSA-B containing 215 mM NaCl (+NaCl), or were cells treated with 1 mg/mL polymyxin B for 80 minutes before being plated on TSA-B (polymyxin B treated). Dilution plating experiments were repeated three times for all strains, and one representative experiment is shown.

bioRxiv preprint doi: https://doi.org/10.1101/2023.04.15.536747; this version posted October 11, 2023. The copyright holder for this preprint (which was not certified by peer review) is the author/funder, who has granted bioRxiv a license to display the preprint in perpetuity. It is made available under aCC-BY 4.0 International license.

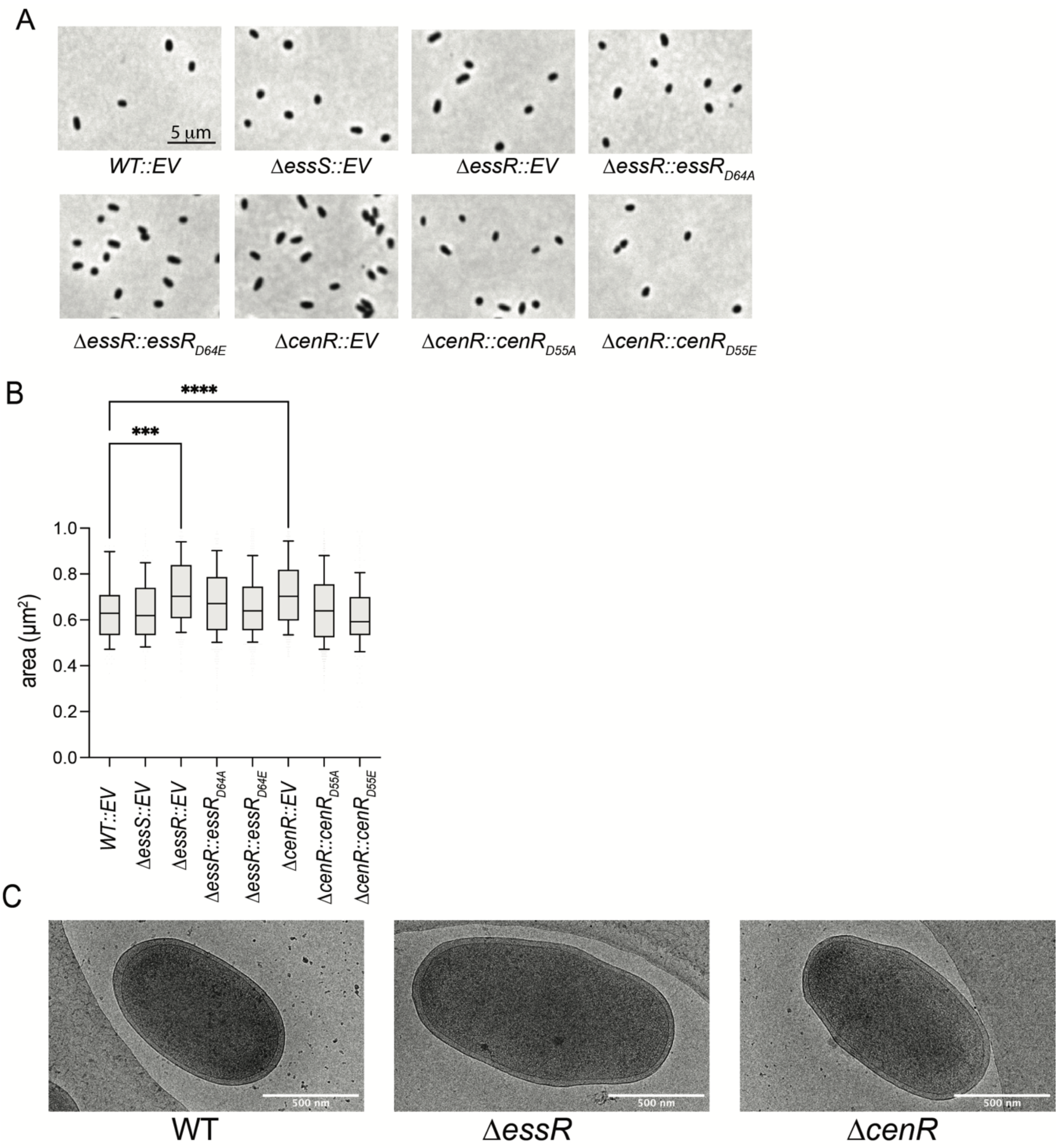

Figure S3: Deletion of *cenR* and *essR* results in increased cell area. A) Phase-contrast micrographs (630x magnification) of WT *B. ovis*, D*essS*, D*essR*, and D*cenR* in-frame deletion mutants; D*essR* complemented with *essR*$_{D64A}$ and *essR*$_{D64E}$; D*cenR* complemented with *cenR*$_{D55A}$ and cen*R*$_{D55E}$. B) Cell area analysis of WT (n=113), D*essS* (n=173), D*essR* (n=242), and D*cenR* (n=535) empty vector control strains (EV), D*essR* complemented with *essR*$_{D64A}$ (n=478) and *essR*$_{D64E}$ (n=466) alleles and D*cenR* complemented with *cenR*$_{D55A}$ (n=908) and cen*R*$_{D55E}$ (n=180) alleles. Mean is shown as a horizontal line in the box (25th-75th percentile); whiskers capture from 10th-90th percentile. Statistical significance was calculated using one-way ANOVA, followed by Dunnett's multiple comparison test to WT empty vector control (WT::EV) ($p < 0.001$, ***; $p < 0.0001$, ****). C) Representative cryo-EM images of WT *B. ovis*, Δ*essR*, and Δ*cenR* in-frame deletion mutants.

bioRxiv preprint doi: https://doi.org/10.1101/2023.04.15.536747; this version posted October 11, 2023. The copyright holder for this preprint (which was not certified by peer review) is the author/funder, who has granted bioRxiv a license to display the preprint in perpetuity. It is made available under aCC-BY 4.0 International license.

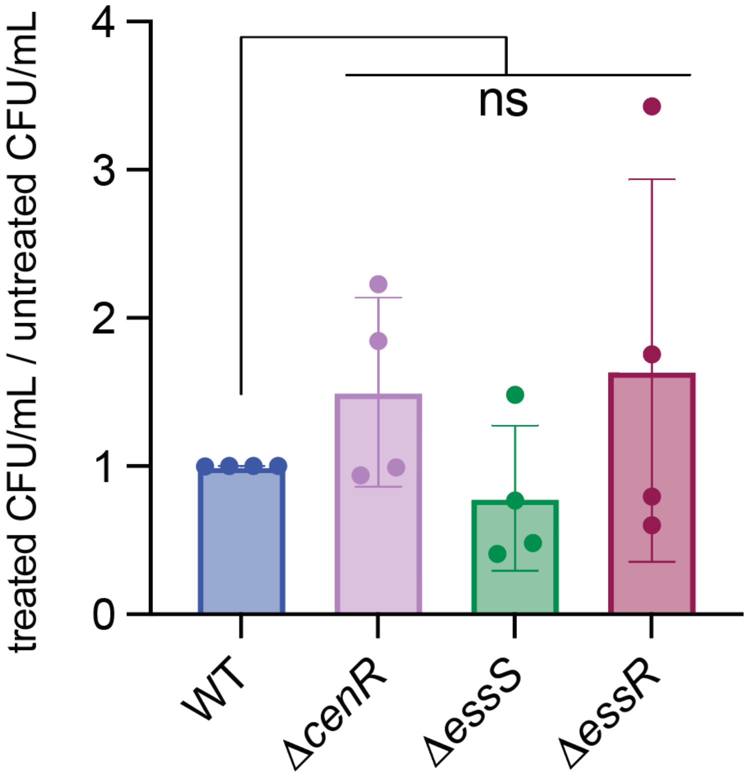

Figure S4: Δ*cenR,* Δ*essS*, and Δ*essR* are not sensitive to acidic pH. Strains harboring in-frame deletions of *cenR, essS*, and *essR* were incubated in Brucella broth at pH 7.0 or Brucella broth at pH 4.2 for 2 h before being serially diluted and plated on TSAB. CFU of treated cultures (pH 4.2) were normalized to CFU of untreated cultures (pH 7). Error bars represent the mean ± standard deviation of the four replicates. Statistical significance was calculated using one-way ANOVA, followed by Dunnett's multiple comparisons test to WT (n.s. = non-significant, $p > 0.05$).

bioRxiv preprint doi: https://doi.org/10.1101/2023.04.15.536747; this version posted October 11, 2023. The copyright holder for this preprint (which was not certified by peer review) is the author/funder, who has granted bioRxiv a license to display the preprint in perpetuity. It is made available under aCC-BY 4.0 International license.

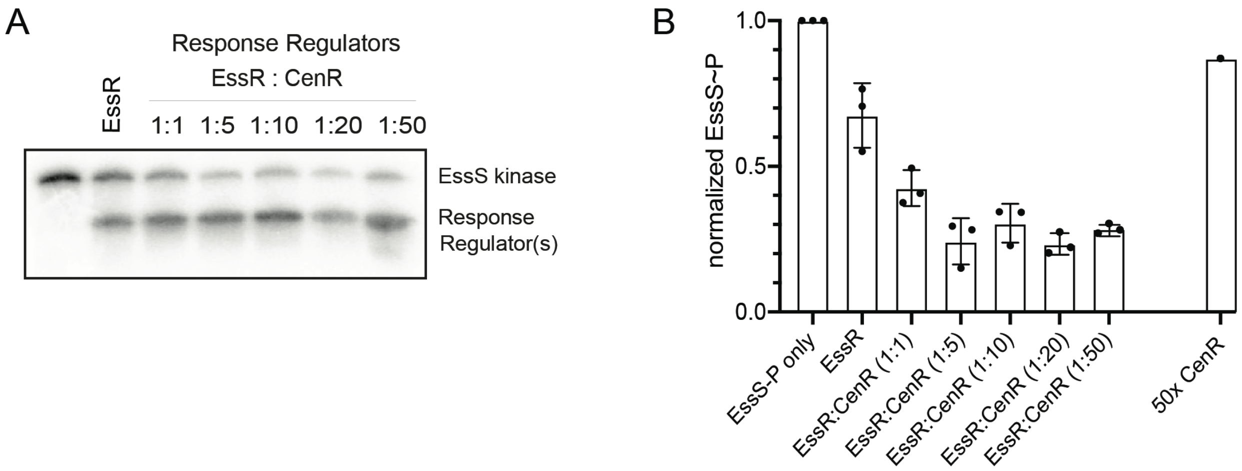

Figure S5. CenR enhances dephosphorylation of EssS~P in the presence of EssR. A) *In vitro* phosphoryl transfer assay with purified EssS kinase domain and EssR (1:1) and increasing ratiometric amounts of CenR. All reactions were stopped after 20s. B) Quantification of EssS~P levels; mean EssS~P band intensity is set to 1 in the EssS~P only reaction. Normalized EssS~P levels 20s after addition of EssR and CenR in varying ratios is plotted. Error bars represent the standard deviation of three biological replicates.

bioRxiv preprint doi: https://doi.org/10.1101/2023.04.15.536747; this version posted October 11, 2023. The copyright holder for this preprint (which was not certified by peer review) is the author/funder, who has granted bioRxiv a license to display the preprint in perpetuity. It is made available under aCC-BY 4.0 International license.

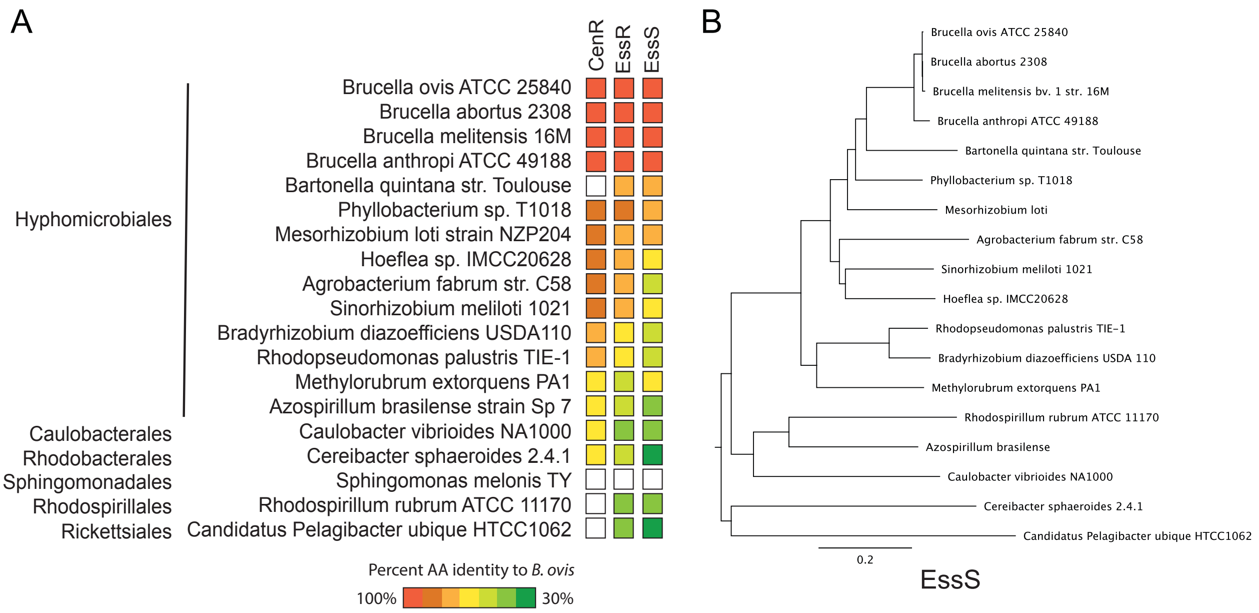

Figure S6: CenR, EssR, and EssS are widely distributed in the class Alphaproteobacteria. A) Representative Alphaproteobacterial genomes were queried for reciprocal top BLAST hits of CenR, EssR, and EssS. A gene was determined as present (colored box) if a reciprocal top-hit pair was found, and as absent (white) if no such pair was found. The color of the boxes reflects the percent amino acid (AA) identity (over the full protein length) to the corresponding *B. ovis* sequences. B) A phylogenetic tree based on the amino acid sequence of EssS orthologs. Global alignment with free end gaps was used as the alignment type and Blosum62 as the cost matrix. Jukes-Cantor was used as the genetic distance model and neighbor-joining was the tree build method with no outgroup.
